# ENC1 Facilitates Colorectal Carcinoma Tumorigenesis and Metastasis via JAK2/STAT5/AKT Axis-Mediated Epithelial Mesenchymal Transition and Stemness

**DOI:** 10.3389/fcell.2021.616887

**Published:** 2021-03-16

**Authors:** Ying Cui, Jiani Yang, Yibing Bai, QingWei Li, Yuanfei Yao, Chao Liu, Feng Wu, Jingchun Zhang, Yanqiao Zhang

**Affiliations:** ^1^Department of Radiation Oncology, Harbin Medical University Cancer Hospital, Harbin, China; ^2^Department of Gastrointestinal Medical Oncology, Harbin Medical University Cancer Hospital, Harbin, China; ^3^Department of Gastroenterology, The First Affiliated Hospital of Harbin Medical University, Harbin, China

**Keywords:** colorectal carcinoma, CRC, ENC1, epithelial mesenchymal transition, stemness

## Abstract

Ectodermal neural cortex 1 (ENC1) is an actin-binding protein and has been known to be upregulated in several cancers, but the molecular mechanisms through which it contributes to the pathology of CRC have largely been elusive. We utilized data mining and validated the aberrant expression of ENC1, following which phenotypic traits of malignancy were assessed *in vitro*. Ruxolitinib was used as a surrogate to compare the effects of ENC1 expression and silencing on the JAK-STAT-AKT pathway. *In vivo* models were employed to confirm the *in vitro* observations. Computation analysis, strengthened by *in situ* and *in vitro* data, confirmed the overexpression of ENC1 in CRC and predicted a poor prognosis, while enhanced cell proliferation, invasion, migration, EMT, and stemness were associated with ENC1 overexpression. Silencing of ENC1 downregulated the phenotypes. Additionally, silencing of ENC1 significantly reduced the activation of JAK2 and consequent activation of STAT5 and AKT comparable to ruxolitinib inhibition of JAK2. Silencing of ENC1 resulted in lesser tumor volumes and fewer numbers of tumors, *in vivo*. These data suggest that ENC1 induces CRC through the JAK2-STAT5-AKT axis. ENC1 is a suitable diagnostic marker for CRC detection, and ENC1 targeting therapies may suppress CRC progression.

## Introduction

A recently concluded longitudinal study undertaken in 195 countries over a period of 27 years (1990–2017) is an in-depth analysis of incidence, mortality, disability, and associated risk factors in Colorectal Cancer Collaborators (CRC) (2019). The study found that CRC is the third most deadly and fourth most found cancer in the world, preceded by lung, liver, and stomach cancer. During the period of the study, there was a consistent increase in the age-standardized incidence rate, and the findings are concurrent with the recent GLOBOCAN database, estimating about 1,800,977 incident cases and a million deaths due to CRC (Bray et al., 2018). These figures are attributed to higher CRC screening and are indicative of a decline or stabilization. However, several countries have been reporting CRC occurrence in individuals under 50 years of age identified with western diet and lifestyle (The Lancet Gastroenterology Hepatology, 2019; Hofseth et al., 2020).

Colorectal cancer is etiologically a heterogeneous disease arising through three major pathways–the traditional adenoma–carcinoma sequence, the serrated pathway, and the inflammatory pathway (Keum and Giovannucci, 2019). A stepwise accumulation of such genetic mutations and/or epigenetic changes is known to contribute to the occurrence of sporadic CRC (Long et al., 2017; Nguyen and Duong, 2018; De Palma et al., 2019). The most implicated molecular pathways in CRC progression are EGFR/MAPK, Notch, PI3K, TGF-β, and Wnt signaling pathways, given the significance of these pathways to critical cellular processes. Further, the cross talk between these pathways in CRC progression has also been discussed (Koveitypour et al., 2019).

The roles of several genes such as EGFR, RAS, RAF, Notch-1, Jagged-1, PIK3CA, PTEN, TGFBR2, TGFBR1, SMADs, AXIN, and CTNNB1 have been assessed and linked to the abovementioned pathways. While reviewing the literature, we identified that several of these genes serve as biomarkers of CRC progression and bear a prognostic value (Garcia-Bilbao et al., 2012). ENC1 is one such gene also found to be overexpressed in primary colon cancers (Garcia-Bilbao et al., 2012; Uddin et al., 2019).

Upregulation of ENC1 has also been recorded in medulloblastoma, prostate, glioblastomas, and astrocytomas, indicating that the gene may have an oncogenic potential if inappropriately expressed (Hammarsund et al., 2004). Expression of ENC1 correlates with the transcriptional activity of the β-catenin/Tcf4 complex and p53 or p53-regulated factors explaining the aberrant expression of ENC1 in CRC (Fujita et al., 2001). However, the molecular role of ENC1 in CRC progression and its role in pathway cross talk remain less explored.

In the current study using a combinatorial approach of *in vitro* and *in vivo* models, we report that ENC1 expression in CRC is associated with increased cellular proliferation, migration, invasion, and tumor growth, and this was likely mediated through the JAK2-STAT5-AKT axis. Through our study, we have elaborated the potential role of ENC1 in primary CRC.

## Materials and Methods

### Data Mining and the Gene Set Enrichment Analysis (GSEA)

The Oncomine database^1^ and GEPIA database^2^ were utilized to assess the mRNA expression status of ENC1 in CRC. From TCGA, we downloaded the gene expression profile data of CRC tissues for a large cohort of CRC patients (*n* = 469) and compared them with normal colon samples (*n* = 41) using bioinformatic tools.

CRC-related RNA-seq data were retrieved from the TCGA database portal^3^ for pathway and function analyses. GSEA software^4^ was applied to calculate enrichment levels. ENC1 expression values were used as the phenotype.

### Tissue Microarrays and Tissue Specimens

The CRC TMAs (HColA180Su17) consisted of 100 primary CRC specimens and 78 matched peritumoral tissues containing complete clinicopathological information and long-term follow-up data which were commercially procured from Shanghai Outdo Biotech Co., Ltd (Shanghai, China).

Twenty-four pairs of snap-frozen CRC specimens and adjacent normal tissues used for quantitative real-time PCR (qRT-PCR) were obtained from the Department of Colorectal Cancer Surgery, the Second Affiliated Hospital of Harbin Medical University, between September 2018 and February 2019. Twelve paired CRC samples were randomly selected for western blotting analysis. Selected patients had only undergone colectomy without neoadjuvant therapy. Pre-approval for the study was sought from the Institutional Review Board of Harbin Medical University.

### Cell Lines and Cell Culture

The human colorectal cancer cell lines HT29, LOVO, DLD-1, SW620, HCT116, SW480, RKO, and normal colorectal cells NCM460 were commercially procured from the ATCC (United States). As per the manufacturer’s instructions, HT29, DLD-1, HCT116, and NCM460 were cultured in RPMI-1640 medium (Gibco, United States) while LOVO was grown in F-12K medium (Gibco, United States) and RKO was in MEM (Gibco, United States), respectively. All cell lines were maintained in a humidified atmosphere with 5% CO_2_ at 37°C. SW620 and SW480 were incubated in Leibovitz’s L-15 medium (Gibco, United States). Uniform, 10% FBS (ScienCell, United States) supplementation without antibiotic was maintained for all cells in the abovementioned medium. All experiments were performed with mycoplasma-free cells. Moreover, all cell lines have been authenticated using STR profiling within the last 3 years.

### Ectopic ENC1 Overexpression and Knockdown of Colorectal Cancer Cells

HT29/HCT116 cells were transiently transfected with plasmid pcDNA3.1 vector as a negative control and full-length pLVX-ENC1-HA (Umibio, Shanghai, China) for ectopic overexpression of ENC1. The transfections were performed by Lipofectamine 2000 (Invitrogen, United States) as per the manufacturer’s protocol. After 48 h of transfection, the following series of experiments were undertaken.

To establish stable ENC1-knockdown cells, HT29/HCT116 cells were infected with lentivirus containing two different shRNAs targeting ENC1 (GeneChem, Shanghai, China). The virus was added to cells cultured in the RPMI-1640 medium at an MOI of approximately 10 with 8 μg/ml polybrene. At 48 h post infection, puromycin (2 μg/mL) was added for an additional 10–14 days to select stable cells. The sequences of shRNA were as follows:

shRNA-negative control: 5′-TTCTCCGAACGTGTCAC GT-3′;
ENC1-shRNA1: 5′-CCATCCACCCAGAAGTCTT-3′;
ENC1-shRNA2: 5′-GCTGATTCCTACTGCATTT-3′.

### Western Blotting

Total proteins were extracted from human tissue samples or cultured cells using RIPA buffer (Solarbio, Beijing, China) containing commercially procured protease and phosphatase inhibitor cocktails (Roche, Mannheim, Germany). The total protein concentration was determined using a BCA kit (Beyotime, shanghai, China). Further, proteins were separated using a 10% SDS-PAGE and then transferred onto PVDF membranes (Merck, Darmstadt, Germany). Upon transfer, membranes were blocked using five percent skimmed milk solution in PBS/Tween-20 for an hour. The membranes were incubated overnight at 4°C with a diluted solution of primary antibodies followed by washing and re-incubation with a horseradish peroxidase (HRP)-conjugated secondary antibody (Zsbio, China) for 1 h at room temperature. The following commercial antibody preparations were used: antibodies for ENC1, E-cadherin, N-cadherin, Vimentin, Snail, CD44, and CD133 were purchased from the Proteintech Group (Wuhan, China); antibodies for p-JAK2 (Tyr1007/1008), JAK2, p-STAT5 (Tyr694), STAT5, p-AKT (Ser473), AKT, and SOX2 were obtained from Cell Signaling Technology (CST, United States); and antibodies for GAPDH (internal controls) were procured from Zsbio, China.

### qRT-PCR

Total RNA was extracted by TRIzol reagent (Thermo Fisher Scientific, United States), and reverse transcription reactions were performed with a ReverTra Ace qPCR RT Master Mix (TOYOBO, Japan). PCR amplification was performed using a SYBR Green Master Mix (Roche, Mannheim, Germany) on an ABI 7500 Fast Real-time PCR Detection System (Applied Biosystems, Foster City, CA, United States). The 2^–ΔΔ*Ct*^ or ΔCt method was selected to calculate relative mRNA expression levels. GAPDH was utilized as the endogenous control to normalize the relative expression levels of target genes. The specific primer sequences used are listed as follows: EN C1-F: 5′-GCAGTAGGAATCAGCGAGTA-3′; ENC1-R: 5′-CC AAGGTGGGAGATGTGA-3′; GAPDH-F: 5′-CATGTTCGTCA TGGGTGTGAA-3′; GAPDH-R: 5′-GGCATGGACTGTGGTCA TGAG-3′.

### Immunohistochemistry (IHC)

For TMA IHC staining, the TMAs were deparaffinized in xylene and rehydrated using graded alcohol after heating at 63°C for an hour, and the DAKO automatic immunohistochemical pretreatment system (Autostainer Link 48, Dako North America, Inc., United States) was used for antigen retrieval. For mouse tumor tissues, all samples were fixed in formalin and paraffin embedded. Briefly, the slides were deparaffinized with xylene and rehydrated using graded ethanol and citric acid under high temperature and pressure was used for antigen retrieval. Then, the TMAs or mouse tissue slices were incubated with the rabbit primary anti-ENC1 (1:150 for TMAs, 1:50 for mouse tumor tissues, ProteinTech, China) and Ki-67 (1:150 for mouse tumor tissues, Zsbio, China) overnight at 4°C. This was followed by goat anti-rabbit IgG secondary antibody for 30 min at room temperature. After washing with PBS, DAB was used for the color reaction and specimens were counterstained with hematoxylin finally.

The immunoreactions were evaluated by two independent researchers blinded to the clinicopathologic information. The staining intensity was categorized as follows: 0 (no staining), 1 (weak), 2 (moderate), and 3 (strong). The positive rate of stained cells was classified as follows: 0 (no positive cells), 1 (<25%), 2 (25–50%), 3 (51–75%), and 4 (76–100%). The immunoreactivity score was calculated by tissue staining intensity multiplied by positive rate of stained cells (range: 0 to 12). Score > 8 was considered as high expression level and ≤8 as low expression level.

### Cell Proliferation Assay

Cancer cells lines (3 × 10^3^ cells per well) were inoculated into 96-well plates in triplicate and incubated at 37°C for 24, 48, 72, and 96 h. CCK-8 reagents (Dojindo, Kumamoto, Japan) with a ratio of 10 μl solution per 100 μl medium were added to each well. Cell viability was measured at the indicated time points, and the absorbance was read at 450 nm under a fluorescence microplate reader.

### Colony Formation Assay

Thousand cells were seeded on the 6-well culture plates with a 10% FBS-containing medium and incubated. After 7 to 14 days, large colonies (>100 cells/per colony) were fixed and stained by methanol and crystal violet for 30 min and counted manually.

### Wound-Healing Assay

Cells were cultured until about 90% confluence in a 6-well plate, then the monolayers were wounded using a 20-μl plastic pipette tip. 0 and 24 h later, cells migrated into the wounded area were photographed. The analysis of wound area was conducted by ImageJ software.

### Transwell Migration and Invasion Assays

For the migration and invasion assays, a total of 5 × 10^5^ cells were seeded in the upper chambers of a transwell plate (Corning, United States) or a Matrigel (BD BioCoat, San Jose, CA, United States)-precoated transwell plate in 200 μl of serum-free medium. The bottom chambers contained 600 μl of RPMI-1640 medium with 20% FBS. After the cells were incubated at 37°C for 48 h, the bottom chambers were fixed by 4% methanol and stained with 1% crystal violet. Before staining, the non-migratory or non-invasive cells on the top surface were gently removed using a cotton swab. Then, cells were randomly counted and photographed with a microscope in at least three fields.

### Immunofluorescence Staining

Cells were seeded in 24-well plates and transfected with plasmids vector and ENC1 until about 95% confluence. After washing thrice with PBS, cells were fixed with 4% paraformaldehyde, permeabilized with 0.2% Triton X-100 (Solarbio, Beijing, China) for 20 min, and blocked with 5% bovine serum albumin for half an hour. The cells were then incubated with primary antibodies against E-cadherin, N-cadherin, and Vimentin (1:100, ProteinTech, China), overnight at 4°C, followed by incubation with corresponding fluorescence-labeled secondary antibodies (1:200, Zsbio, China) at room temperature for 1 h. The nuclei were stained with 4′-6-diamidino-2-phenylindole (DAPI; Beyotime, shanghai, China) for 15 min. Fluoroscent microscope-guided imaging was undertaken.

### Sphere Formation Assay

A total of 3 × 10^3^ cancer cells were plated in ultra-low-attachment 6-well culture plates (Corning, United States) containing DMEM/F-12 (Gibco, United States) supplemented with B-27 supplement (Gibco, United States), 20 ng/ml human EGF (PeproTech, Israel), and 20 ng/ml human FGF (PeproTech, Israel). After 10 to 14 days of incubation at 37°C, the tumor stem spheres were imaged and the number of spheres ≥50 μm was counted under a light microscope.

### Flow Cytometry Analysis

The concentration of cancer cells was adjusted to 1 × 10^6^/ml. The cells were washed three times with ice-cold PBS and incubated with FITC-labeled mouse anti-human CD44 antibody (BD Biosciences, 560977) for 30 min in the dark and then analyzed using a flow cytometer instrument (BD Biosciences).

### *In vivo* Studies

All mice used in studies were BALB/c nude mice (4–5 weeks old, female) purchased from Vital River Laboratory (Beijing, China), were maintained under a constant temperature and humidity under pathogen-free conditions with a 12-h light–dark cycle, and received free access to food and water. For the xenograft mouse model, HCT116 cells transfected with ENC1 shRNA-1 and Ctrl-shRNA (5 × 10^6^, suspended in 100 μl of PBS) were inoculated into the left dorsal flank of mice that randomized into two groups (*n* = 6). The tumor volumes were measured using calipers every 4 days and calculated using the equation: (length × width^2^)/2. After the last measurement of tumor volume, the mice were humanely sacrificed by carbon dioxide asphyxiation, cervical dislocation was performed subsequently to ensure death, then tumor tissues were removed and used for subsequent assays.

For the lung metastasis mouse model, mice (2 mice/group) were given intravenous tail vein injections of 5 × 10^6^ HCT116 cells (ENC1 shRNA-1, Ctrl-shRNA). About 10 weeks later, the mice were euthanized (the method was administered as above), and lungs were harvested and imaged. The lung tissue was collected in paraformaldehyde for further H&E staining and IHC analysis. All animal studies were taken at the animal laboratory of the Second Affiliated Hospital of Harbin Medical University and approved by the Ethics Committee for Animal Experimentation of Harbin Medical University.

### Statistical Analysis

All data from at least three independent experiments were presented as the mean ± SD. GraphPad Prism 7.0 statistical software (GraphPad, San Diego, CA, United States) and SigmaPlot 14.0 (Systat Software, San Jose, CA, United States) were used for the two-tailed Student’s *t*-test, Wilcoxon matched-pair test, analysis of variance (ANOVA), Tukey’s post test, and chi-square test, when appropriate. The Kaplan–Meier method was used to draw survival curves, and the log-rank was performed for statistical analysis. Univariate and multivariate survival analyses were conducted using a Cox proportional hazard regression model. Values of *P* < 0.05 were considered statistically significant (^∗^), *P* < 0.01 was considered more statistically significant (^∗∗^), and *P* < 0.001 was considered the most statistically significant (^∗∗∗^).

## Results

### High ENC1 Expression in CRC Correlates With Poor Prognosis

**FIGURE 1:**
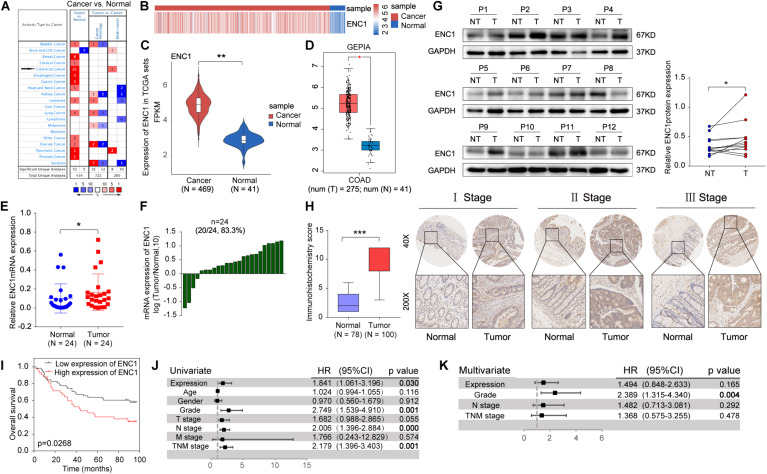
ENC1 expression is often upregulated in CRC and associated with poor clinical outcomes. **(A)** The ENC1 mRNA expression levels in CRC and other cancers from the Oncomine cohort. The threshold was selected with the following parameters: p-value of 1E-4, fold change of 2, and gene rank of top 10%. **(B,C)** Heat map and Violin plot presented the upregulated expression of ENC1 between CRC (*n* = 469) and normal colon tissues (*n* = 41) by bioinformatics analysis from TCGA. Unpaired *t*-test, ** *P* < 0.01. **(D)** ENC1 expression was overexpressed in CRC (*n* = 275) compared with normal colon tissues (*n* = 41) by box plot in the GEPIA database. *P < 0.05. **(E)** The ENC1 mRNA expression in 24 paired CRC patients quantified from qRT-PCR. Wilcoxon matched-pair test, *P < 0.05. **(F)** Log10-fold change was used to show the differential mRNA expression of ENC1 between CRC and normal tissues. **(G)** The ENC1 protein expression was detected by Western blotting in 12 pairs of CRC tissues (T) and adjacent normal tissues (NT). The scatter plot revealed the relevant density of ENC1 protein expression. **(H)** IHC analysis of ENC1 expression in CRC TMAs. Representative stage images were shown (upper: magnification × 40, scale bar = 500 μm; lower: magnification × 200, scale bar = 100 μm). Box plot described the IHC score of ENC1 in 100 CRC tissues (IHC score: 9.17 ± 2.719) and 78 matched peritumoral tissues (IHC score: 2.397 ± 1.59). Mean ± SD, unpaired *t*-test, ***P < 0.001. **(I)** Kaplan–Meier’s overall survival curve showed that the patients with a higher expression level of ENC1 have a shorter survival period. **(J)** Univariate and **(K)** multivariate Cox proportional hazard analyses were conducted to evaluate the HR of ENC1 for overall survival in CRC.

To investigate the relationship between ENC1 expression and clinical significance in CRC, we first utilized data mining from public databases to analyze the ENC1 expression status. Based on the Oncomine database, the result showed significantly increased ENC1 mRNA expression patterns in 21 CRC datasets (Figure 1A). Following which, differential expression of ENC1 between CRC and normal colon tissues was analyzed through published files. We downloaded the gene expression profile data of CRC from TCGA which contains 469 CRC tissues and 41 adjacent normal colon tissues. Heat map and Violin plot exhibited the ENC1 expression which was markedly upregulated in CRC contrasted with normal colon tissues (Figures 1B,C). Meanwhile, data from GEPIA corroborated with those from the TCGA cohort substantiating enhanced ENC1 expression in CRC tissues (*n* = 275) in comparison to normal tissues (*n* = 41) (Figure 1D).

To further confirm the results from computation analysis, we examined the mRNA expression levels of ENC1 in 24 paired CRC patients using qRT-PCR. We found that ENC1 was significantly overexpressed in 20/24 (83.3%) of CRC samples (Figures 1E,F). Moreover, the increased ENC1 protein expression was detected in 12 pairs of CRCs compared with normal tissues by Western blotting (Figure 1G). Based on CRC TMAs, 100 CRC patients were tested by IHC. Figure 1H shows representative ENC1 images of immunostaining from patients in different clinical stages. Cytoplasmic ENC1 immunoreactivity was higher in tumors than in matched adjacent normal tissues. Additionally, box plots illustrated that the IHC score of ENC1 was upregulated in CRC samples. Findings from our cohort were identical to findings from other public databases.

The relationship between ENC1 expression and clinicopathologic characteristics was also explored. The clinicopathological features in 100 CRC patients with informative IHC data are categorized in Table 1, including age, gender, grade, T stage, N stage, M stage, and TNM stage. Based on the staining score, the results revealed that higher ENC1 expression was positively associated with T stage (*P* = 0.020). Additionally, Kaplan–Meier’s overall survival analysis described that the patients with higher ENC1 expression level associated with poorer survival in CRC (*p* = 0.0268; Figure 1I). Furthermore, univariate COX proportional hazard analysis demonstrated that high levels of ENC1, Grade, N stage, and TNM stage were significantly associated with worse survival of CRC patients (Figure 1J). Multivariate Cox proportional hazard analysis indicated that Grade (*P* = 0.004) was an independent prognostic factor for poor survival of CRC patients (Figure 1K). By univariate Cox model analysis, we found that the increased level of ENC1 in CRC was a risk factor of prognosis, but further multivariate analysis implicated that this risk factor bore statistical significance. The above findings are suggestive that ENC1 is upregulated and may serve as a considerable prognostic indicator for CRC patients.

**TABLE 1:** Correlation between ENC1 expression and clinicopathological features in 100 CRC patients.

	Variables	ENC1 expression		Total	χ^2^	*p* value
		
		Low	High			
Age (year)					0.134	0.714
	≤65	27	18	45		
	>65	31	24	55		
Gender					0.164	0.685
	Female	28	22	50		
	Male	30	20	50		
Grade					1.588	0.208
	I/II	51	33	84		
	III/IV	7	9	16		
T stage					5.429	**0.020**
	T1/T2/T3	51	29	80		
	T4	7	13	20		
N stage					0.066	0.797
	N0	36	25	61		
	N1/N2	22	17	39		
M stage					1.395	0.238
	M0	58	41	99		
	M1	0	1	1		
TNM stage					0.066	0.797
	I/II	36	25	61		
	III/IV	22	17	39		

*The bold value is statistically significant (*p* < 0.05).*

### ENC1 Overexpression or Knockdown Affects Cell Proliferation, Migration, and Invasion of CRC *in vitro*

**FIGURE 2:**
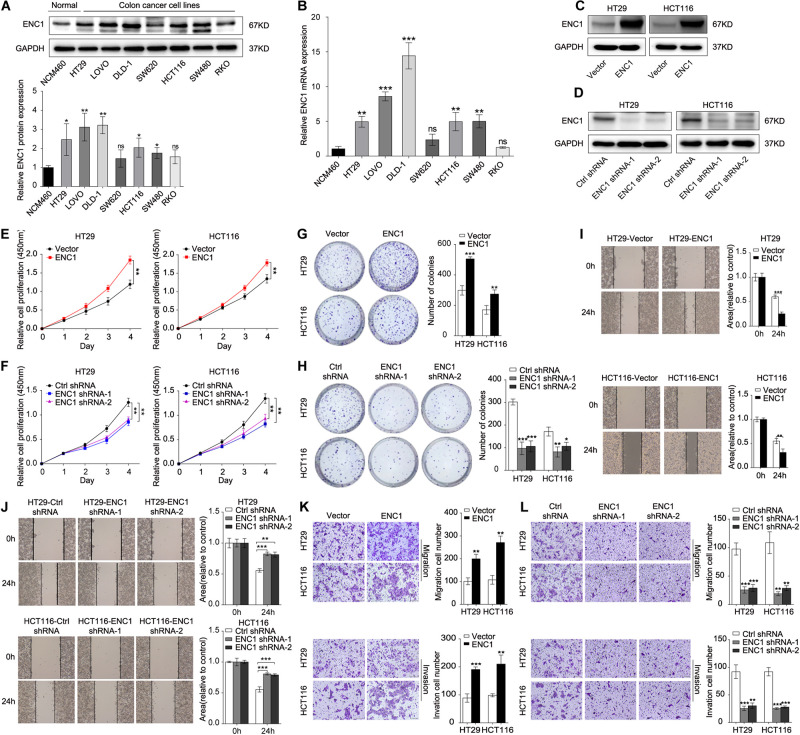
ENC1 regulates proliferation, migration, and invasion of CRC cells. **(A)** Endogenous ENC1 protein expression status was tested in human normal colonic epithelial cells (NCM460) and CRC cells (HT29, LOVO, DLD-1, SW620, HCT116, SW480, and RKO) by Western blotting. **(B)** Endogenous ENC1 mRNA expression status was compared between NCM460 and CRC cells by qRT-PCR. Western blot analysis of ENC1 protein expression levels in ENC1 overexpression **(C)** and ENC1 knockdown **(D)** CRC cells HT29/HCT116. **(E,F)** CCK-8 and **(G,H)** colony formation assays were performed to assess cell proliferation in CRC cells with ENC1 overexpression or knockdown. Wound-healing assay was used to detect cell migration ability in ENC1 overexpression **(I)** and ENC1 knockdown **(J)** CRC cells. Transwell assays showed the effect of ENC1 overexpression **(K)** and ENC1 knockdown **(L)** on CRC cell migration and invasion (left: representative images; right: quantitative analyses). Results are presented as the mean ± SD of three independent experiments. *P < 0.05, **P < 0.01, and ***P < 0.001.

To excavate the underlying biological behaviors of ENC1 in CRC, we evaluated endogenous ENC1 expression levels in seven CRC cell lines (HT29, LOVO, DLD-1, SW620, HCT116, SW480, and RKO) compared with the human normal colonic epithelial cell line (NCM460) by western blotting and qRT-PCR. The protein and mRNA expression patterns of ENC1 were significantly higher in five CRC cell lines (HT29, LOVO, DLD-1, HCT116, and SW480) than normal (Figures 2A,B). HT29/HCT116 was selected for both gain of function and loss of function simultaneously in subsequent experiments because of their moderate levels of expression.

Furthermore, we identified functions between low and high ENC1 expressions on TCGA by GSEA, and GO-REGULATION-OF-DENDRITIC-CELL-DIFFERENTIATION (cell differentiation) was enriched in the ENC1 high group (Supplementary Figure 1A; *P* = 0.06679). Although the result was not statistically significant, it is deemed to represent a trend. Next, to verify this hypothesis, we performed a series of experiments to evaluate whether the suggested signatures may be involved *in vitro*. We ectopically overexpressed ENC1 to gain of function and used two ENC1-targeting shRNAs (shRNA1 and shRNA2) to loss of function in HT29/HCT116 cells. ENC1 protein expression levels were significantly increased in ENC1 overexpression and decreased in ENC1 knockdown CRC cells as assessed by Western blotting (Figures 2C,D and Supplementary Figures 1B,C). CCK-8 and colony formation assays revealed that overexpression of ENC1 significantly accelerated the CRC cell proliferation (Figure 2E) and colony formation ability (Figure 2G). In contrast, after the silencing of ENC1, growth, and colony formation numbers of cells were attenuated (Figures 2F,H). Furthermore, we investigated the impact of ENC1 on cell migratory and invasive properties in HT29/HCT116. Wound-healing assay reflected that overexpression of ENC1 resulted in faster closing of scratch wounds whereas ENC1 depletion resulted in delayed wound closure (Figures 2I,J). As shown in Figure 2K, migration and invasion abilities were also confirmed through migration and Matrigel invasion assays in an ENC1-overexpressing cell population. Contrarily reduced cell motility and invasive capability were observed in the ENC1-silenced cell type (Figure 2L). The results from these analyses illustrated that ENC1 modulates CRC cell tumorigenesis and progression *in vitro*.

### ENC1 Accelerates the EMT Process and Maintains Stemness Phenotypes of CRC

**FIGURE 3:**
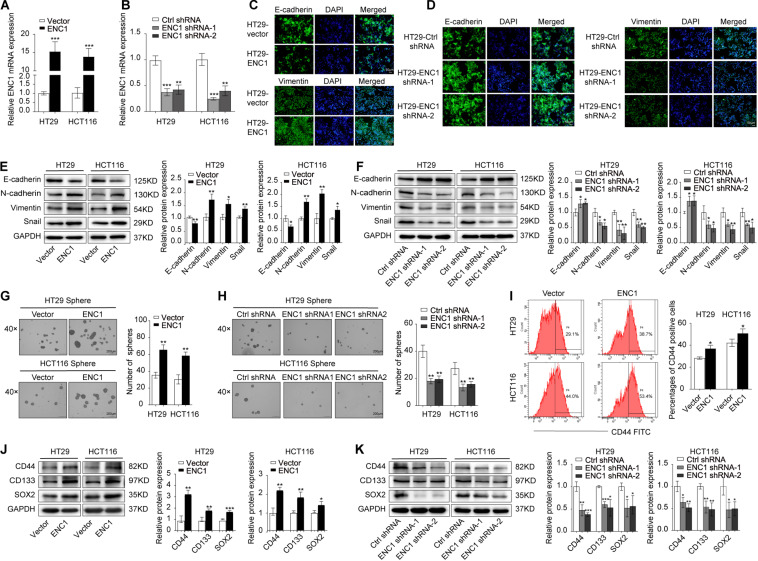
ENC1 overexpression induces EMT and maintains stemness of CRC. **(A,B)** The ENC1 overexpression or knockdown efficiency was assessed in ENC1-overexpression and ENC1-knockdown HT29/HCT116 cells by qRT-PCR. **(C,D)** Immunofluorescence staining of E-cadherin and Vimentin expression in overexpressed or silenced HT29 (magnification × 20, scale bar = 50 μm), and the cell nuclei were stained with DAPI in blue. **(E,F)** EMT markers of E-cadherin, N-cadherin, Vimentin, and Snail protein expression levels in ENC1-overexpression and ENC1-knockdown CRC cells were detected by Western blotting. Representative images of sphere formation and sphere number analysis were displayed (magnification × 40, scale bar = 200 μm) in HT29/HCT116 after ENC1 overexpression **(G)** and ENC1 knockdown **(H)**. **(I)** Flow cytometry analysis of CD44-positive cells in the indicated groups of cells. **(J,K)** Western blotting analysis of stemness markers (CD44, CD133, and SOX2) expression in ENC1-overexpression and ENC1-knockdown CRC cells. Results are presented as the mean ± SD of three independent experiments. *P < 0.05, **P < 0.01, and ***P < 0.001.

Deciphered using the framework developed previously, it has been widely known that EMT and cancer stemness are closely related to the tumor progression and metastasis in various types of cancer cells (Pang et al., 2017; Matsuyama et al., 2019; Park et al., 2019a; Wu et al., 2020). Thus, we speculated that ENC1 might be involved in the EMT process and stemness in CRC cells. To evidence our speculation, immunofluorescence staining, sphere formation assay, and flow cytometry analysis of CD44, well-known EMT, and stemness-related markers by Western blotting were further determined. First, the overexpression or knockdown efficiency of ENC1 in HT29/HCT116 cells was validated by qRT-PCR (Figures 3A,B). Next, to confirm EMT, immunofluorescence staining assay reflected that ENC1 overexpression induced a weaker E-cadherin (epithelial marker) and stronger Vimentin (mesenchymal marker) expression in HT29 cells (Figure 3C), while ENC1 silencing reversed the phenotype (Figure 3D). The changes of representative EMT markers (E-cadherin, N-cadherin, Vimentin, and Snail) were verified through western blotting subsequently. As illustrated in Figure 3E, overexpression of ENC1 led to increased levels of N-cadherin, Vimentin, and Snail but lessened the levels of E-cadherin in HT29/HCT116. Expectedly, the observations were reversed upon ENC1 silencing (Figure 3F).

To confirm the impact on stemness of HT29/HCT116 cells, we examined tumor sphere-forming ability that directly correlated with cancer stemness. More and larger spheres formed in ENC1-overexpressed cells (Figure 3G), while fewer and less spheres were seen in ENC1-silenced cells (Figure 3H) compared with controls in CRC. We also assessed the cell surface marker CD44 expression levels using flow cytometry. As shown in Figure 3I, the ectopic expression of ENC1 upregulated the proportion of CD-44-positive cells in HT29/HCT116. Finally, we assessed the effect of ENC1 on sentinel markers associated with stemness, including CD44, CD133, and SOX2 in CRC cells by western blotting. We observed conspicuous increased protein levels of CD44, CD133, and SOX2 by ENC1 overexpression (Figure 3J) and declined protein level by ENC1 knockdown (Figure 3K). Taken together, data implicated that there is a positive correlation and regulation of ENC1 in EMT progress and maintenance of stemness.

### ENC1 Knockdown Eliminates Xenograft Tumor Growth and Lung Metastasis *in vivo*

**FIGURE 4:**
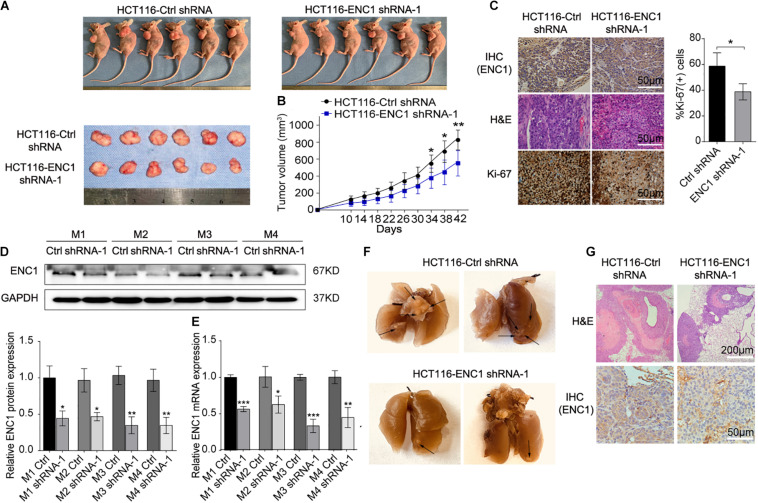
*In vivo*, downregulation of ENC1 inhibits xenograft tumor growth and lung metastasis. **(A)** Images of xenograft tumors from six BALB/c nude mice at 42 days after subcutaneously inoculated with HCT116-Ctrl shRNA and HCT116-ENC1 shRNA-1 cells. **(B)** The growth of xenograft tumors was measured by tumor volume every 4 days and tumor growth curves were plotted between the comparison in two groups (*n* = 6). **(C)** Representative images of IHC and H&E staining show that ENC1 silencing downregulated the expression of ENC1 and Ki-67 in xenograft tumors (magnification × 400, scale bar = 50 μm). The protein and mRNA levels of ENC1 were also analyzed in xenograft tumor tissues by Western blotting **(D)** and qRT-PCR **(E)**, respectively. **(F)** Representative images of metastatic lungs in the HCT116-Ctrl shRNA (left) group and HCT116-ENC1 shRNA-1 (right) group. Black arrows indicated the metastatic pulmonary nodules. **(G)** H&E staining (upper panel: magnification × 100, scale bar = 200 μm) and IHC staining with ENC1 (lower panel: magnification × 400, scale bar = 50 μm) were performed on section of metastatic pulmonary nodules. Results are presented as the mean ± SD of three independent experiments. Student’s *t* test was applied for statistical analysis in two-group comparison: *P < 0.05, **P < 0.01, and ***P < 0.001.

In addition to validate above findings *in vivo*, we constructed mouse xenograft and lung metastasis models. The mouse xenograft model was established to prove whether silenced ENC1 could retard tumor growth *in vivo*. After subcutaneous injection with HCT116-ENC1 shRNA-1 and HCT116-Ctrl-shRNA into the left dorsal flank of nude mice, tumor volume was monitored by a caliper for 6 weeks. When the mouse tumor size in the ENC1 shRNA-1 group was significantly less than the Ctrl-shRNA group, xenograft tumors were isolated and imaged (Figure 4A). The changes in tumor growth were plotted on a tumor growth curve which showed that mouse tumor size decreased in the ENC1 shRNA-1 group (Figure 4B). Additionally, H&E staining results identified tumor morphological characteristics and IHC showed reduced the expression of ENC-1 and Ki-67 in the Ctrl-shRNA group in comparison to xenograft tumor groups (Figure 4C). Western blotting (Figure 4D) and qRT-PCR (Figure 4E) verified ENC1 protein and mRNA expression being downregulated in the ENC1 shRNA-1 xenograft tumor group.

In order to verify whether tumors of lung metastasis were restricted by ENC1 shRNA-1 *in vivo*, mice were given intravenous tail vein injections of HCT116-ENC1 shRNA-1 and HCT116-Ctrl-shRNA, respectively. Ten weeks after injection, the mice were sacrificed and lungs were imaged. As represented in Figure 4F, the mice injected with HCT116-ENC1 shRNA-1 cells formed fewer nodules on the lung surfaces than mice injected with HCT116-Ctrl-shRNA cells. Metastatic nodules on the surfaces of mice lungs were confirmed by H&E staining, whereas the expression level of ENC1 in the nodules was also verified by IHC staining (Figure 4G), thus concluding that ENC1 knockdown restrains xenograft tumor growth and lung metastasis.

### ENC1 Modulates the JAK2/STAT5/AKT Axis in CRC

**FIGURE 5:**
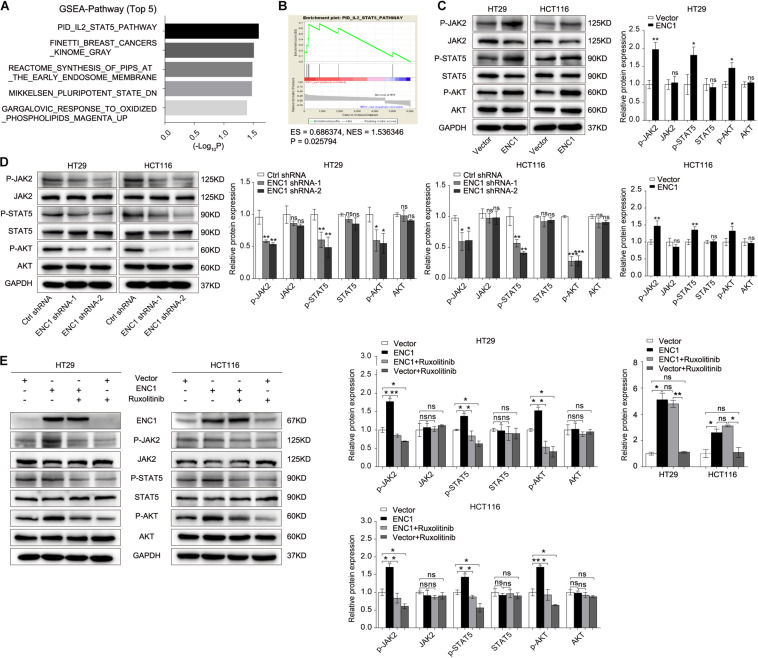
ENC1 activates JAK2/STAT5/AKT signaling in CRC. **(A)** Top five significantly and positively enriched pathways with high ENC1 expression by GSEA. **(B)** ENC1 expression positively correlated with STAT5-activated gene signatures (PID_IL2_STAT5_PATHWAY). **(C,D)** Western blotting analysis of JAK2/STAT5 signaling key members (p-JAK2, p-STAT5, p-AKT, JAK2, STAT5, and AKT) in HT29/HCT116 after ENC1 overexpression and ENC1 knockdown. **(E)** ENC1-overexpressed HT29/HCT116 were treated with JAK1/2 inhibitor ruxolitinib. After being treated for 24 h, as indicated, p-JAK2, p-STAT5, p-AKT, JAK2, STAT5, and AKT expression levels were detected by western blotting. Results are presented as the mean ± SD of three independent experiments. *P < 0.05, **P < 0.01, and ***P < 0.001.

To dissect the potential molecular mechanisms mediated by ENC1 in CRC tumorigenesis and progression, we performed GSEA for pathway enrichment in the TCGA CRC database to identify biological pathways between low and high ENC1 expressions. Figure 5A shows the most significantly and positively enriched pathways of the top five with a high ENC1 expression phenotype, and the STAT5-related pathway (PID_IL2_STAT5_PATHWAY) indicated the most meaningful association with ENC1 (ES = 0.686374, NES = 1.536346, *P* = 0.025794) (Figure 5B). As has been previously established, abnormal activation of JAK2/STAT5 signaling led to cell proliferation, differentiation, and other biological processes in human cancers (Gu et al., 2013; Jiang et al., 2019; Wang et al., 2020). Thus, we focused to assess the effects of ENC1 on JAK2/STAT5 signaling. Aiming to confirm the functional gene signatures, we detected the effects of ENC1 on the protein levels of JAK2/STAT5 signaling target genes by Western blotting. We found that the phosphorylation levels of JAK2, STAT5, and AKT increased in ENC1-overexpressed CRC cells (Figure 5C) but decreased in ENC1-silenced CRC cells (Figure 5D). However, no considerable changes were observed in total JAK2, STAT5, and AKT protein levels from different panels (Figures 5C,D).

Ruxolitinib is a selective JAK1 and JAK2 inhibitor that targets JAK/STAT-associated signaling, exhibiting an effective clinical treatment of myelofibrosis adopted by FDA (Chang et al., 2019), which was used to confirm the effect of ENC1 on activation of the JAK2/STAT5/AKT axis in CRC cells. HT29/HCT116 cells after transfection with ENC1 and empty vector were treated with a concentration of 25 μM ruxolitinib for 24 h which prevented the release of activated JAK2. Predictably, the upregulated expression levels of phosphorylated JAK2, STAT5, and AKT induced by ENC1 overexpression effectively reversed with ruxolitinib treatment. However, ruxolitinib suppressed ENC1-induced JAK2 release and STAT5 and AKT expression but did not affect ENC1 expression and total JAK2, STAT5, and AKT protein levels in HT29/HCT116 cells (Figure 5E). We thus present the significance of ENC1 in activating the JAK2/STAT5/AKT axis.

### ENC1 Promotes CRC Cell Tumorigenesis, Progression, EMT, and Stemness Through Activating the JAK2/STAT5/AKT Axis

**FIGURE 6:**
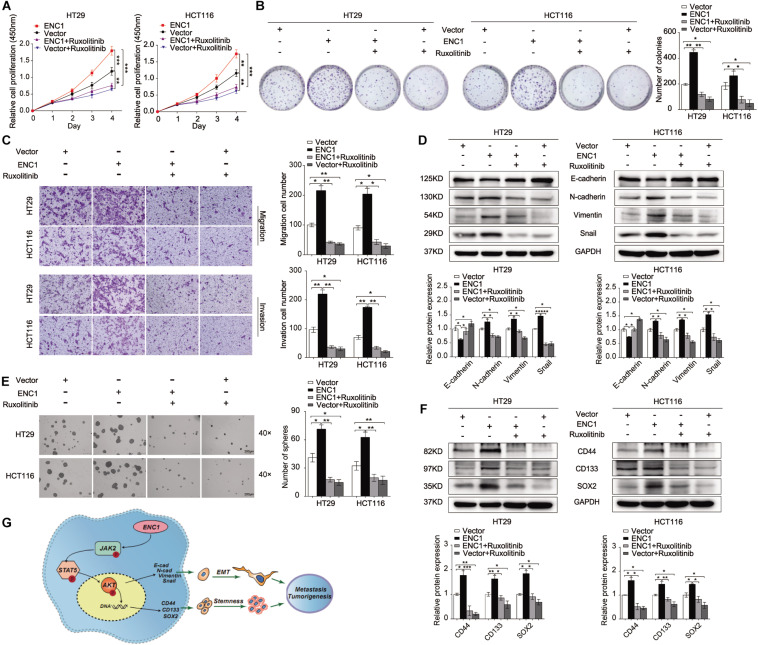
ENC1 accelerates cell proliferation, migration, invasion, EMT, and stemness through activating JAK2/STAT5/AKT signaling in CRC. Cell proliferation was assessed by CCK8 assay **(A)** and colony formation assay **(B)** in ENC1-overexpression CRC cells treated with ruxolitinib. **(C)** Effects of ruxolitinib treatment on ENC1-overexpressed HT29/HCT116 cell motility and invasiveness capability were detected by Transwell assay. **(D)** Western blotting showed the protein changes of EMT markers between ENC1-overexpressed cells with and without ruxolitinib treatment. **(E)** Effect of ruxolitinib in tumor spheres caused by ENC1 overexpression in contrast with no treatment. **(F)** The expression of stemness markers (CD44, CD133, SOX2) were detected by Western blotting in ENC1-overexpressed CRC cells treated with ruxolitinib. **(G)** Schematic diagram of ENC1 promotes CRC tumorigenesis and metastasis via the JAK2/STAT5/AKT axis mediated EMT and stemness. Results are presented as the mean ± SD of three independent experiments. *P < 0.05, **P < 0.01, and ***P < 0.001.

To further clarify the oncogenic effects of ENC1 on JAK2/STAT5/AKT axis activation in tumorigenesis and metastasis properties of CRC, CCK-8, and colony formation assays were first examined to assess the cell proliferation with or without ruxolitinib treatment. As shown in Figures 6A,B, ruxolitinib treatment weakened the proliferation and colony formation ability of ENC1-overexpressed CRC cells and less inhibition were observed in cells without ectopic expression. Transwell assays revealed that the enhanced effects of ENC1 on HT29/HCT116 cell motility and invasiveness capability were also alleviated with ruxolitinib treatment (Figure 6C).

Additionally, we assessed the role of the ENC1-activated JAK2/STAT5/AKT axis in EMT and stemness. Sphere formation assay was undertaken, and EMT and stemness-related markers were assessed by Western blotting. Notably, ruxolitinib could restrict tumor-sphere formation caused by ENC1 overexpression (Figure 6E). EMT markers (E-cadherin, N-cadherin, Vimentin, and Snail) along with stemness markers (CD44, CD133, and SOX2) were significantly less pronounced, indicating that ruxolitinib mediated the disruption of ENC1 effects in CRC cells (Figures 6D,F). Briefly, blockage of JAK2/STAT5 signaling could effectively prevent CRC cell tumorigenesis, progression, EMT, and stemness mediated by ENC1.

## Discussion

Western lifestyles and diet have been identified as substantial risks to the development and progression of colorectal cancer (CRC), the burden of which is faced most by low- and middle-income countries. The global estimates for cancers worldwide report an increasing trend of both incidence and mortality in countries of the Baltic, Russia, and China (Bray et al., 2018). While early and more effective screening is helpful in reducing the burden of CRC, early onset of the disease and changing molecular paradigms present a challenge to diagnosis and existing successful therapies.

A common focus in cancer disease diagnosis is a search for robust molecular markers that would facilitate both diagnosis and prognosis. Similar approaches have been applied to CRC, and the utility of several genes and microRNAs with strong upregulation in CRC has been explored (Garcia-Bilbao et al., 2012; Pellino et al., 2018; Alves Martins et al., 2019). One such candidate gene with limited exploration in CRC biology is ENC1 (Garcia-Bilbao et al., 2012). ENC1 encodes for an actin-associated protein. The expression of ENC1 was found high during gastrulation in the neuroectodermal region of the epiblast and later in the nervous system. Normally, it has been ascribed to have roles in neuronal and adipocyte differentiation; however, the aberrant expression of ENC1 has been reported in human brain tumors including glioblastoma and astrocytoma (Kim et al., 2000). Further, ENC1 dysregulation and associated malignancies have been confirmed in cancers of prostrate (Lapointe et al., 2004), ovarian (Fan et al., 2019), pancreatic (Ross et al., 2000), and colon (Fujita et al., 2001). While some studies have a cause–effect link, some identify the molecular mechanisms that drive the activation of ENC1 (Kim et al., 2000; Fujita et al., 2001). Very few studies identify the pathway downstream of ENC1 and its contribution to transformation. Our study is the preliminary demonstration of a detailed analysis of pathways downstream of ENC1 contributing to malignancy in a CRC model. The study is a triage presenting data from computational, *in vitro*, and *in vivo* experimental analyses. By a comparison of various databases, we compared the expression profile of ENC1 across CRC and normal patients. This computational analysis was validated with CRC tissues and normal adjacent samples using qRT-PCR and western blotting methods. We also investigated the correlation between ENC1 expression and clinical outcomes by IHC data, high expression of ENC1 related to advanced T stage, and unfavorable clinical outcomes in patients with CRC. The expression was further confirmed in CRC cell lines, and analysis is further carried out using HT29 and HCT 116 confirming the phenotypic malignancy traits of increased cell proliferation, migration, invasion, EMT, sphere formation, and stemness. ENC1 induced EMT, stemness, and other prognostic features described above, and its association with clinical prognosis, to the best of our knowledge, has not been discussed previously. Thus, the study is a significant advancement contributing to the advancement of understanding of CRC at a molecular level and utilizing the phenotypic changes for prognostic implications.

Further, we hypothesized that these individual phenotypic traits may have a common master regulator activated by ENC1, which in turn would regulate the multiple pathways at transcriptional and translational levels. The role of the JAK-STAT pathway in EMT and stemness has been discussed (Jin, 2020). For instance, single-cell cultures of cells derived from myxoid liposarcoma expressed different amounts of canonical JAK-STAT transcripts (Dolatabadi et al., 2019). Similar studies exploring the roles of JAK-STAT signaling and induction of EMT and cancer stemness have been demonstrated for hematopoietic cancers (Wingelhofer et al., 2018), oral squamous cell carcinoma (Chen et al., 2020), and colon cancers (Park et al., 2019b), among others. The role of EMT effectors (Snail, Slug among others) on EMT core regulators (Vimentin, E-cadherin, and N-cadherin) through modulation of various signaling pathways and epigenetic regulation of EMT effectors has been discussed previously (Vu and Datta, 2017; Huang et al., 2020). However, the role of ENC1 as a modulator of EMT effectors and stemness via the JAK-STAT pathway has not been reported previously.

Our GSEA analysis on the TCGA database indicated that a high expression of ENC1 was associated with the JAK2-STAT5-AKT axis, thereby supporting our hypothesis. While, the expression of ENC1 positively correlated with JAK2-STAT5 and AKT, the activation of JAK2 via STAT5 remains elusive. Since JAK2 activation can result from ligation of several homo- and hetero-dimeric pairs of signaling receptors, belonging to class I and II cytokine receptor families, we thus propose two alternative mechanisms of activation of JAK 2 in CRC via the ENC1 pathway. (1) ENC1 is an actin-binding protein like Src homology 2 (SH2) domain-containing adapter protein SH2B1β which is known to bind to JAK2 and potentiate its activation in response to other proteins such as growth hormone or leptin (Rider and Diakonova, 2011). (2) Evidence has also been mounting for several non-canonical methods of JAK activation wherein a subset of receptors exists in a pre-dimerized state and conformational shifts in the transmembrane domain result in JAK activation (Ferrao et al., 2018). It is also likely that ENC1 through alternative pathways leads to activation of cytokines and/or growth hormones which canonically activate the JAK-STAT pathway. However, the hypothesis tests bearing.

In conclusion, our study is the first report demonstrating the downstream ENC1-JAK2-STAT5-AKT axis and its implications on CRC progression and metastasis. It is also a demonstration of how ENC1 activation may not only contribute to the adenomatous carcinoma and the immunological pathway through the JAK-STAT pathway. The study is a novel proposition with silencing of ENC1, not only significantly reducing the malignant phenotype but also showing a therapeutic reduction in tumor numbers and volumes *in vivo.*

The utility of ENC1 as a diagnostic, prognostic, or even plausible therapeutic target is proposed. A complete understanding of the suitability and feasibility of the proposed work may benefit resource strengthening and allocation at a global scale.

## Data Availability Statement

The datasets presented in this study can be found in online repositories. The names of the repository/repositories and accession number(s) can be found in the article/Supplementary Material.

## Ethics Statement

The studies involving human participants were reviewed and approved by Committees for the Institutional Review Board of Harbin Medical University, China. The patients/participants provided their written informed consent to participate in this study. The animal study was reviewed and approved by Committees for the Institutional Review Board of Harbin Medical University, China. Written informed consent was obtained from the owners for the participation of their animals in this study.

## Author Contributions

YC conceived and performed the experiments, analyzed the data, and wrote the manuscript. JY and YB carried out the experiments and analyzed the data. QL, FW, and JZ were involved in the implementation of the study. YZ and YY guided the study and corrected the manuscript. CL participated in the project guidance. All authors contributed to the article and approved the submitted version.

## Conflict of Interest

The authors declare that the research was conducted in the absence of any commercial or financial relationships that could be construed as a potential conflict of interest.

## Footnotes

1
https://www.oncomine.org/

2
http://gepia.cancer-pku.cn/

3
https://cancergenome.nih.gov/

4
http://www.broadinstitute.org/gsea
