# Corrigendum: ENC1 Facilitates Colorectal Carcinoma Tumorigenesis and Metastasis via JAK2/STAT5/AKT Axis-Mediated Epithelial Mesenchymal Transition and Stemness

**DOI:** 10.3389/fcell.2021.758671

**Published:** 2021-09-03

**Authors:** Ying Cui, Jiani Yang, Yibing Bai, QingWei Li, Yuanfei Yao, Chao Liu, Feng Wu, Jingchun Zhang, Yanqiao Zhang

**Affiliations:** ^1^Department of Radiation Oncology, Harbin Medical University Cancer Hospital, Harbin, China; ^2^Department of Gastrointestinal Medical Oncology, Harbin Medical University Cancer Hospital, Harbin, China; ^3^Department of Gastroenterology, The First Affiliated Hospital of Harbin Medical University, Harbin, China

**Keywords:** colorectal carcinoma, CRC, ENC1, epithelial mesenchymal transition, stemness

In the published article, there was an error in the affiliations. The affiliation “Translational Medicine Research and Cooperation Center of Northern China, Heilongjiang Academy of Medical Sciences, Harbin, China” was mistakenly included. The correct affiliations should be as follows “1 Department of Radiation Oncology, Harbin Medical University Cancer Hospital, Harbin, China; 2 Department of Gastrointestinal Medical Oncology, Harbin Medical University Cancer Hospital, Harbin, China; 3 Department of Gastroenterology, The First Affiliated Hospital of Harbin Medical University, Harbin, China.”

The authors apologize for this error and state that this does not change the scientific conclusions of the article in any way. The original article has been updated.

## Publisher's Note

All claims expressed in this article are solely those of the authors and do not necessarily represent those of their affiliated organizations, or those of the publisher, the editors and the reviewers. Any product that may be evaluated in this article, or claim that may be made by its manufacturer, is not guaranteed or endorsed by the publisher.

