# Supplementary material for: ENC1 Facilitates Colorectal Carcinoma Tumorigenesis and Metastasis via JAK2/STAT5/AKT Axis-Mediated Epithelial Mesenchymal Transition and Stemness
Source: Front Cell Dev Biol. 2021 Mar 16;9:616887. doi: 10.3389/fcell.2021.616887 (PMC8010667; doi:10.3389/fcell.2021.616887)
Supplement: Supplementary file 1 [file Table_1.DOCX]

**Supplementary Figures**


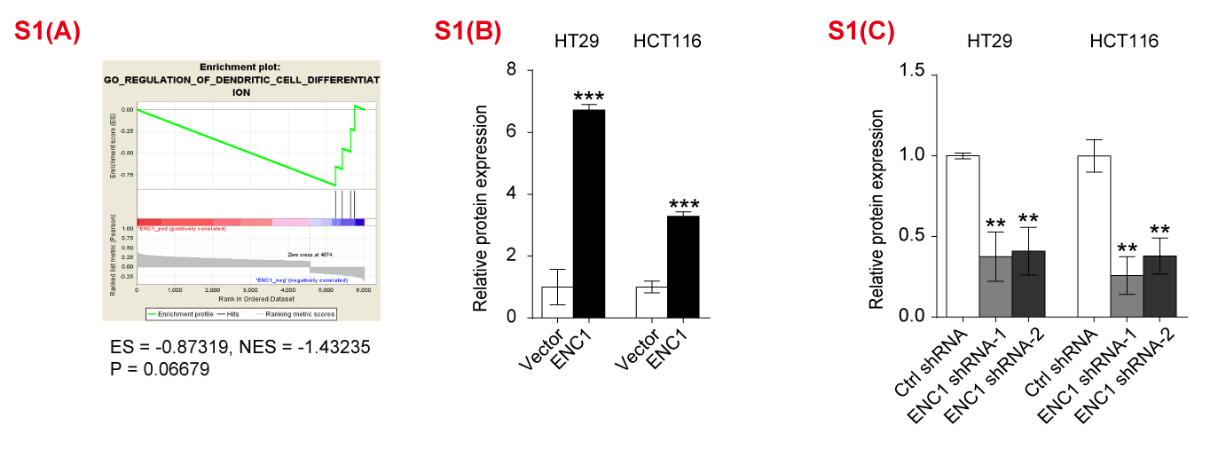


**Supplementary Figure. S1(A)** (Related to Fig. 2) GSEA showed that ENC1 expression negatively correlated with cell differentiation activated gene signatures (GO-REGULATION-OF-DENDRITIC-CELL-DIFFERENTIATION) (p = 0.06679). **FIGURE. S1(B, C)** (Related to Fig. 2C, D) The gray value analyses of ENC1‐overexpression and ENC1‐knockdown efficiency in HT29/HCT116.
